# Use of quantum dots of sizes magic in biological systems

**DOI:** 10.1186/1753-6561-8-S4-P258

**Published:** 2014-10-01

**Authors:** Samantha Deus, Felipe Luz, Anielle Silva, Noelio Dantas, Marcelo Silva

**Affiliations:** 1 Instituto de Genética e Bioquímica (INGEB), Universidade Federal de Uberlândia, Santa Mônica, Uberlândia, MG, Brazil; 2 Instituto de Ciências Biomédicas (ICBIM), Universidade Federal de Uberlândia, Santa Mônica, Uberlândia, MG, Brazil; 3 Instituto de Física (INFIS), Universidade Federal de Uberlândia, Santa Mônica, Uberlândia, MG, Brazil

## 

QDs are inorganic semiconductor nanocrystals with quantum confinement and physical and chemical properties different from their counterparts. These properties are controlled depending on the size, shape and structure of nanocrystals, which can absorb and emit a predetermined range of the electromagnetic spectrum. QDs measure 2-10 nm but was recently created the MSNs QDs (nanocrystals sizes magicians), measuring 1-2 nm with well-defined structures, high stability during and after the growth, the presence of a few unit cells in their composition, show strong quantum confinement and different thermodynamically stable structures. In biomarkers, MSNs QDs have several advantages over organic dyes: exhibit high molar absorption absorption spectrum continuous, high intensity luminance and high stability. Therefore QDs may be used in nanomedicine detection of cancers. Once injected into the body dialing UV indicate the location of the tumor devices that capture this fluorescence particle, greatly improving diagnostic procedures. Therefore, the objective is to improve the QDs MSNs for use in biological systems. Laboratory of New Insulating Materials and Semiconductors (LNMIS) were synthesized, probably for the first time. These QDs were tested in HeLa cells and Ehrlich. The results showed that are stable after 4 years the synthesis. In vitro assays showed high fluorescence scattered throughout the cytoplasm, demonstrated by double-labeling with DAPI, up to 20 hours after incorporation by the cell. Thus, incorporation of these QDs by tumor cells and their stability allows them to be used in studies of tumor cell migration in vivo.

## References

[B1] Chen X Samia ACS Lou Y Burda C Investigation of the Crystallization Process in 2 nm CdSe Quantum Dots J Am Chem Soc 2005 127 4372 4375 10.1021/ja0458219 15783219

[B2] Mcbride JR Dukes AD III Schreuder MA Rosenthal SJ On ultrasmallnanocrystals Chem Phy Lett 2010 498 1 9 10.1016/j.cplett.2010.08.052 PMC2994270 21132106

[B3] Nurbosyn J Zhanpeisov U Fukumura H Baba Y Rajan MI Structure Property Correlation of CdSe Clusters Using Experimental Results and First-Principles DFT Calculations J Am Chem Soc 2006 128 629 636 10.1021/ja0565018 16402851

[B4] Soloviev VN Eichhofer A Fenske D Banin U Molecular Limit of a Bulk Semiconductor: Size Dependence of the "Band Gap" in CdSe Cluster Molecules J Am Chem Soc 2000 122 2673 2674 10.1021/ja9940367

[B5] Ute RG Markus G Sara CJ Roland N Thomas N Quantum dots versus organic dyes as fluorescent labels Nature Methods 2008 5 9 763 775 10.1038/nmeth.1248 18756197

